# Supplementary material for: Iterative improvement in the automatic modular design of robot swarms
Source: PeerJ Comput Sci. 2020 Dec 7;6:e322. doi: 10.7717/peerj-cs.322 (PMC7924708; doi:10.7717/peerj-cs.322)
Supplement: Supplemental Information 3 [file peerj-cs-06-322-s003.zip › argos3/doc/api/standalone/a00643_source.html]

ARGoS: plugins/simulator/visualizations/qt-opengl/qtopengl\_lua\_editor.h Source File


- Main Page
- Related Pages
- Namespaces
- Classes
- Files

- File List
- File Members

# plugins/simulator/visualizations/qt-opengl/qtopengl\_lua\_editor.h

Go to the documentation of this file.

```
00001 
00007 #ifndef QTOPENGL_LUA_EDITOR_H
00008 #define QTOPENGL_LUA_EDITOR_H
00009 
00010 namespace argos {
00011    class CQTOpenGLLuaEditor;
00012 }
00013 
00014 #include <QPlainTextEdit>
00015 
00016 namespace argos {
00017 
00018    /****************************************/
00019    /****************************************/
00020 
00021    class CQTOpenGLLuaEditor : public QPlainTextEdit {
00022 
00023       Q_OBJECT
00024 
00025    public:
00026 
00027       CQTOpenGLLuaEditor(QWidget* pc_parent);
00028       virtual ~CQTOpenGLLuaEditor() {}
00029       
00030       void LineNumberAreaPaintEvent(QPaintEvent* pc_event);
00031       int LineNumberAreaWidth();
00032 
00033    protected:
00034 
00035       void resizeEvent(QResizeEvent* pc_event);
00036 
00037    private slots:
00038 
00039       void UpdateLineNumberAreaWidth(int);
00040       void HighlightCurrentLine();
00041       void UpdateLineNumberArea(const QRect& c_rect, int n_dy);
00042 
00043    private:
00044 
00045       /********************/
00046       /********************/
00047 
00048       class CLineNumberArea : public QWidget {
00049 
00050       public:
00051          CLineNumberArea(CQTOpenGLLuaEditor* pc_editor) :
00052             QWidget(pc_editor) {
00053             m_pcEditor = pc_editor;
00054          }
00055 
00056          QSize sizeHint() const {
00057             return QSize(m_pcEditor->LineNumberAreaWidth(), 0);
00058          }
00059 
00060       protected:
00061 
00062          void paintEvent(QPaintEvent* pc_event) {
00063             m_pcEditor->LineNumberAreaPaintEvent(pc_event);
00064          }
00065 
00066       private:
00067 
00068          CQTOpenGLLuaEditor* m_pcEditor;
00069 
00070       };
00071 
00072       /********************/
00073       /********************/
00074 
00075       CLineNumberArea* m_pcLineNumberArea;
00076 
00077    };
00078 
00079 }
00080 
00081 #endif
```

---

Generated on 10 Jul 2018 for ARGoS by 
 1.6.1
